# Reduction of Parasitic Capacitance of A PDMS Capacitive Force Sensor

**DOI:** 10.3390/mi9110570

**Published:** 2018-11-03

**Authors:** Tatsuho Nagatomo, Norihisa Miki

**Affiliations:** 1 School of Integrated Design Engineering, Keio University, Yokohama 223-8522, Japan; tatsuho19950307@keio.jp; 2 Department of Mechanical Engineering, Keio University, Yokohama 223-8522, Japan

**Keywords:** polydimethylsiloxane, parasitic capacitance, ultraviolet treatment, capacitive force sensor

## Abstract

Polymer-based flexible micro electro mechanical systems (MEMS) tactile sensors have been widely studied for a variety of applications, such as medical and robot fields. The small size and flexibility are of great advantage in terms of accurate measurement and safety. Polydimethylsiloxane (PDMS) is often used as the flexible structural material. However, the sensors are likely subject to large parasitic capacitance noise. The smaller dielectric constant leads to smaller influences of parasitic capacitance and a larger signal-to-noise ratio. In this study, the sensor underwent ultraviolet (UV) exposure, which changes Si–CH_3_ bonds in PDMS to Si–O, makes PDMS nanoporous, and leads to a low dielectric constant. In addition, we achieved further reduction of the dielectric constant of PDMS by washing it with an ethanol–toluene buffer solution after UV exposure. This simple but effective method can be readily applicable to improve the signal-to-noise ratio of PDMS-based flexible capacitive sensors. In this study, we propose reduction techniques for the dielectric constant of PDMS and applications for flexible capacitive force sensors.

## 1. Introduction

Polymer-based flexible micro electro mechanical systems (MEMS) tactile sensors have been widely studied for a variety of applications, such as medical and robotics fields [1,2,3,4,5,6,7,8,9,10,11]. The small size and flexibility are of great advantage in terms of accurate measurement and safety. This trend is further accelerated by the electrodes that are robust against deformation. For example, graphene is a promising candidate and the sensors with graphene electrodes were reported along with their fabrication technologies [7,8]. Liquid metal is another candidate, which can form the electrodes by being filled up inside microchannels [9,10,11]. We reported flexible MEMS sensors made of polydimethyl siloxane (PDMS) structural layers and three-dimensional liquid metal electrodes to detect both normal and shear force [10,11].

PDMS is in many cases used as the structural material due to its good mechanical properties and ease of microfabrication. As is often the case with PDMS-based MEMS sensors, the parasitic capacitance originating from a short distance between wirings deteriorates the signal-to-noise ratio. In previous work, parasitic capacitance was compensated for by the electrical circuit [12,13] and low-dielectric constant materials were inserted between the wirings [14]. However, this technique drastically increases the complexity of the fabrication processes and the inserted material may deteriorate the flexibility. 

In this work, to reduce parasitic capacitance, we attempted to reduce the dielectric constant of PDMS layers. PDMS is a low-k material and is known to decrease the dielectric constant after being exposed to ultraviolet (UV), which breaks up Si–CH_3_ to increase Si–O. This topographic change makes the surface of low-k materials nanoporous, which culminates in a reduction of their dielectric constant [15,16]. In addition, we attempted to remove the unbridged materials by chemical washing in PDMS in order to make it further nanoporous. Washing PDMS with the ethanol-hexane buffer solution is known to make PDMS nanoporous [17]. We characterized the combination of these two techniques, i.e., UV-treatment and chemical washing (we used ethanol-toluene), for the first time and demonstrated reduction of the parasitic capacitance of PDMS-based sensors to enhance the signal-to-noise ratio (SNR) using the flexible sensors we developed in our prior work [11]. The techniques are simple and easily applicable to the PDMS layers, which will be stacked to form three-dimensional flexible MEMS sensors with low parasitic capacitance.

## 2. Theory and Method

### 2.1. Theory

#### 2.1.1. Parasitic Capacitance

Parasitic capacitances are the unexpected capacitance generated between the electrical parts in a circuit. Especially in MEMS devices, the electrical parts are close together because of the small size, and therefore parasitic capacitances are difficult to ignore. Inserting low dielectric constant materials between the electrical parts was reported to be effective to reduce the parasitic capacitance [14]. 

#### 2.1.2. Ultraviolet (UV) Treatment on Polydimethylsiloxane (PDMS) Porous Dielectrics

**Figure 1:**
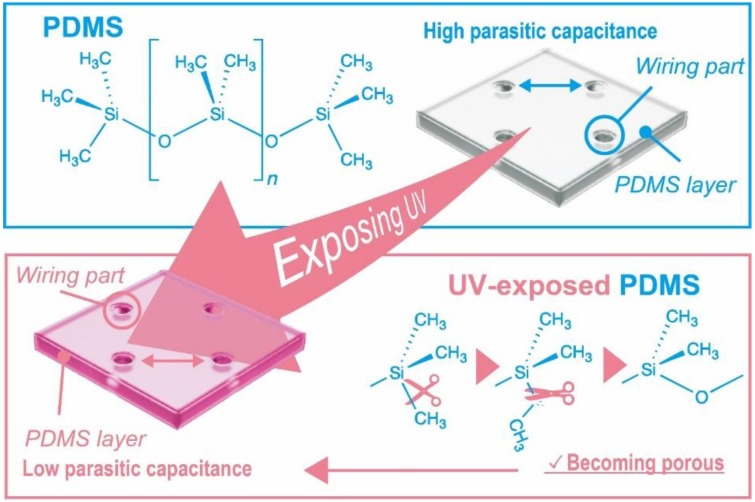
High parasitic capacitance caused by the wiring. The wiring will be formed inside the holes seen in the figure, which will be filled with a conductive liquid metal. Ultraviolet (UV) treatment of polydimethylsiloxane (PDMS) reduces the dielectric constant and parasitic capacitance.

Martinez confirmed the behavior of low-k film under ultraviolet cure and measured its dielectric constant [16]. UV cure treatment breaks Si–CH_3_ bonds and relinks Si–O bonds on the surface of low-k film, leaving nanopores. The electric constant of low-k materials has permittivity under 2.5; however, it is still higher than that of air. Therefore, nanoporous low-k films have a lower dielectric constant than nontreated low-k films. PDMS has the same Si–CH_3_ bonds as low-k materials. PDMS is widely used as a substrate for flexible MEMS devices. We considered that UV cure treatment would lower the dielectric constant of PDMS, as shown in Figure 1, resulting in smaller parasitic capacitance.

### 2.2. Method

#### 2.2.1. UV Treatment on PDMS Membranes

**Figure 2:**
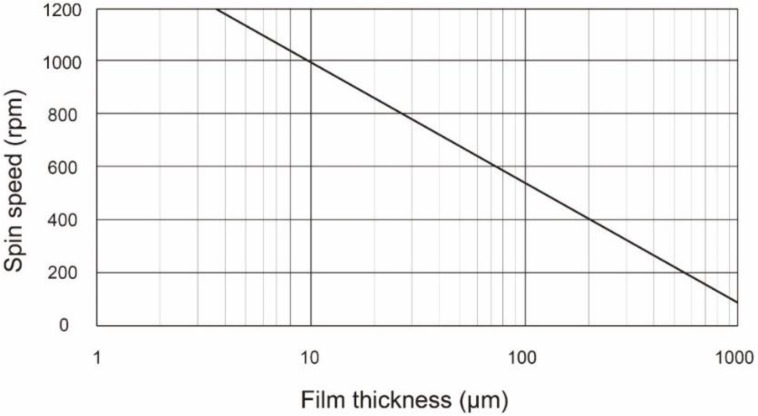
The relationship between spinning speed and film thickness was obtained based on a power law.

First, we fabricated PDMS membranes (Silpot 184 W/C, Dow Corning Toray Co., Ltd., Tokyo, Japan) which were spin-coated on a glass substrate with a spin coater (1H-D7, Mikasa Corporation, Hiroshima, Japan). The ratio of main agent to curing agent was 10:1. The tested film thicknesses were 250, 500, 750, and 1000 μm. Figure 2 shows the relationship between spin time and film thickness. This relationship was empirically obtained based on a power law [18,19,20]. After peeling off the PDMS membrane, we measured the capacitance of two parallel copper plates sandwiching each PDMS membrane by using an LCR meter (ZM2376, NF Corporation, Yokohama, Japan). The applied voltage and frequency were 1.0 V and 1.0 kHz, respectively. The dielectric constant was deduced from the measured capacitance.

Second, we exposed each PDMS membrane to UV (wavelength λ = 405 nm) for 0 to 60 s, which corresponded to 15,600 μW/m^2^. After UV treatment, the dielectric constant of each membrane was deduced using the above-mentioned method. 

#### 2.2.2. Washing of PDMS Membranes

Removing unbridged materials from PDMS further reduced the dielectric constant. We washed PDMS with a buffer solution. We formed a 250-μm thick PDMS membrane and exposed it to UV for 300 s, which corresponded to 78,000 μW/m^2^. Subsequently, we washed the PDMS membrane for 10 s in the buffer solution, which was a mixture of ethanol (ethanol 99.5%, Fujifilm Wako Pure Chemical Corporation, Osaka, Japan) and toluene (toluene 99.5%, Fujifilm Wako Pure Chemical Corporation, Osaka, Japan) at a ratio of 10:1. The dielectric constant of the treated PDMS membrane was measured. We tested 250-μm thick membranes since it showed good results in reduction of the parasitic capacitance by UV-treatment as will be described in Section 3.1.1.

#### 2.2.3. Periods While the Low Dielectric Constant of PDMS Membrane was Maintained

We experimentally investigated how long the low dielectric constant of PDMS membrane was maintained after treatment. We tested the UV-treated membranes and the washed UV-treated membranes. We measured the dielectric constant for 1 week with an LCR meter. The applied voltage and frequency were 1.0 V and 1.0 kHz, respectively. 

#### 2.2.4. Fabrication Process of Capacitive Force Sensor

**Figure 3:**
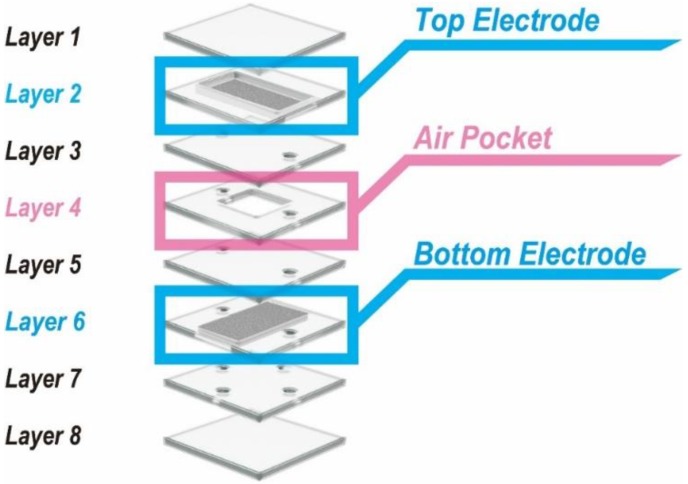
Structure of capacitive force sensor.

Figure 3 shows a schematic image of the capacitive force sensor, which consisted of two parallel-plate electrodes and eight layers of liquid metal and PDMS. When a force was applied to the top of the sensor, the air pocket was deformed and the distance between the electrodes changes, thus the capacitance changes. The applied force can be deduced from the variation of capacitance.

**Figure 4:**
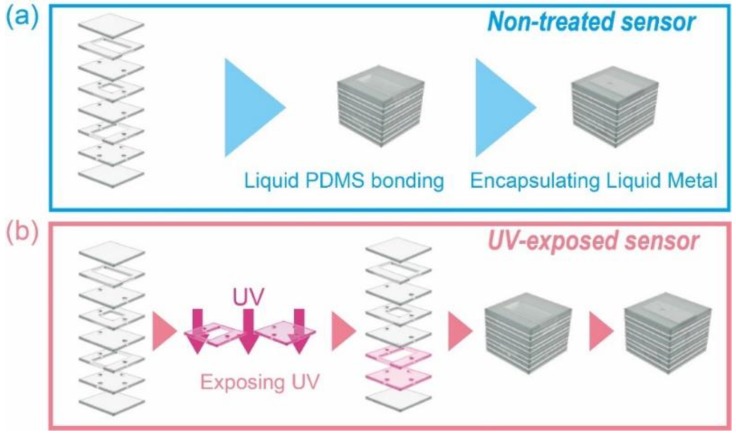
Fabrication process of (**a**) nontreated sensor and (**b**) UV-exposed sensor.

**Figure 5:**
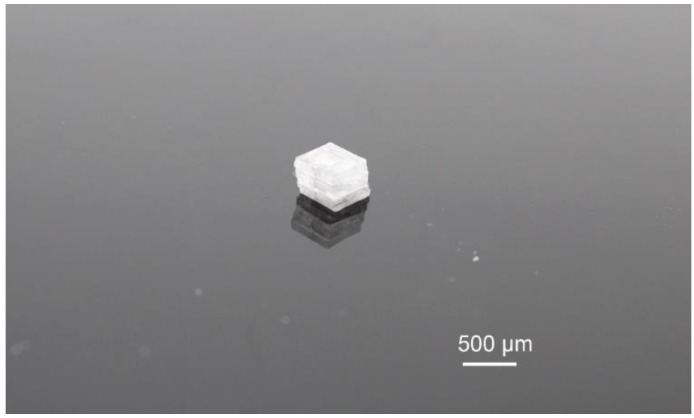
Photograph of the fabricated sensor.

The fabrication process is succinctly illustrated in Figure 4a. First, PDMS was poured into a poly methyl methacrylate (PMMA) mold, which was fabricated by a numerical control (NC) cutting machine (MM-100, Modia Systems Co., Saitama, Japan), and cured at 65 °C for 6 h. PDMS layers were peeled off from the molds and bonded to each other via liquid PDMS [21]; the liquid PDMS (PDMS and toluene in a 2:3 mixture) was spin-coated on a glass substrate and the bonding interface of the PDMS layers were contacted to have the liquid PDMS on the surface prior to bonding. This was cured at 65 °C for 2 h. Galinstan, a liquid metal composed of 68.5% gallium, 21.5% indium, and 10% tin, was injected into these channels. The fabricated sensor is shown in Figure 5.

Layers 6 and 7 had electrical parts that were close together. Therefore, the parasitic capacitance needed to be considered. These layers were exposed to UV light and then washed. After we fabricated PDMS layers, layers 6 and 7 were exposed to UV for 300 s (15,600–15,596 μW/m^2^). We washed these layers in the buffer solution. Whole PDMS layers were bonded to each other via liquid PDMS. This was cured at 65 °C for 2 h, and finally, liquid metal was injected into these channels. 

#### 2.2.5. Comparison with UV-Washed, UV-Treated, and Noncoated Sensors

**Figure 6:**
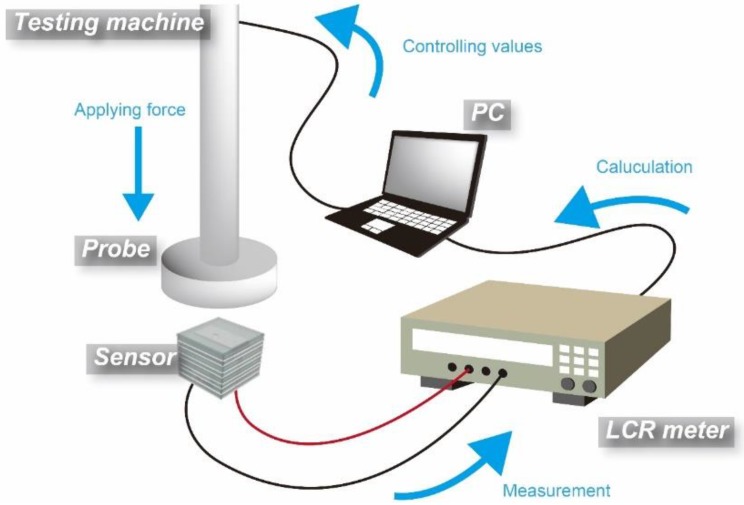
Experimental setup for measurement of parasitic capacitance.

We applied a force to the manufactured sensor of up to 1 N at a speed of 0.1 N/s using a compression machine (Micro Autograph MST-I, Shimadzu Corporation, Kyoto, Japan). The capacitance change was measured, and the influence of the parasitic capacitance was calculated using the SN ratio. In this study, we obtained the SN ratio based on 0-N–type characteristics of the Taguchi method [22]. The measurement conditions were a voltage of 1.0 V and a frequency of 1.0 kHz. This experimental setup is illustrated in Figure 6.

## 3. Results and Discussion

### 3.1. Change of the Dielectric Constant

#### 3.1.1. Effect of UV Treatment

**Figure 7:**
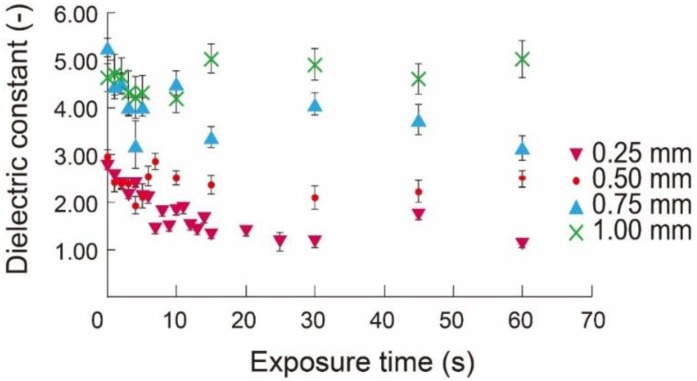
Dielectric constant of PDMS layers with respect to UV treatment time (n = 20).

Figure 7 shows the change of the dielectric constant with respect to UV exposure time. For the PDMS membrane that was 250 μm thick, a decrease of the dielectric constant was observed up to the UV exposure time of 30 s, after which the dielectric constant did not show further reduction. The PDMS membranes thicker than 500 μm showed the lowest dielectric constant after treatment for 5 s, after which the dielectric constant recovered and plateaued. These results indicate that the thin 250-μm PDMS membrane could be effectively treated using UV, while the effects became small for the thicker membranes. In low-k materials, structural changes using UV treatment were observed at the surface [15]. Therefore, the thinner PDMS membrane is more effectively modified with a larger surface/volume ratio. 

#### 3.1.2. Effect of Washing

**Figure 8:**
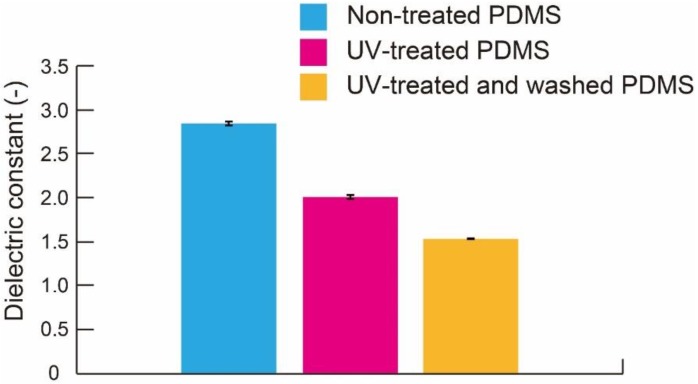
Dielectric constant of nontreated PDMS, UV-treated PDMS, and washed PDMS membranes (n = 20).

We investigated the effect of washing the UV-treated PDMS membrane, as shown in Figure 8. The dielectric constant of nontreated PDMS, UV-treated PDMS, and UV-treated and washed PDMS is 2.85, 1.98, and 1.53, respectively. This result verifies the effectiveness of the washing process. It removed the unbridged materials in PDMS and promoted the formation of nanopores. 

#### 3.1.3. Stability of the Reduced Dielectric Constant of PDMS Membranes

**Figure 9:**
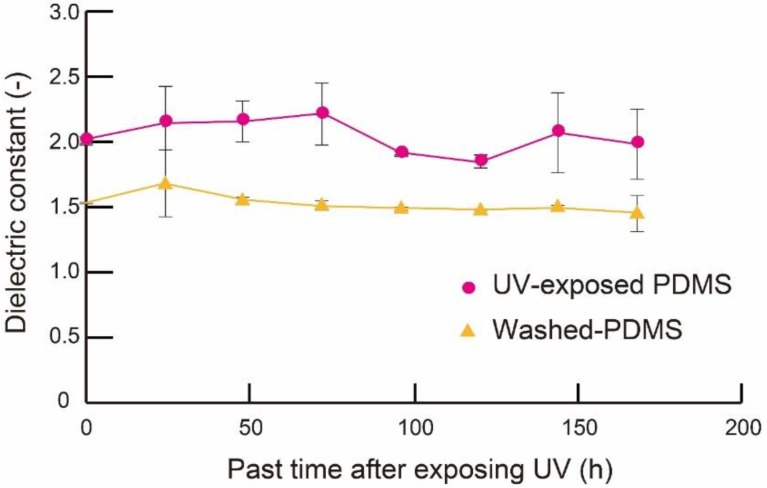
Dielectric constant of a 250-μm thick PDMS layer after UV treatment with or without washing (n = 20).

We investigated the long-term stability of the effects of UV treatment and the combination of UV treatment and washing. The PDMS membrane that was 250 μm thick was exposed to UV light for 300 s. Half the samples were washed with a buffer solution for 10 s. The resulting change of the dielectric constant with respect to time is shown in Figure 9. Both samples maintained the reduced dielectric constant for 168 h, which indicates that the chemical reaction caused by UV treatment had good long-term stability. In washed PDMS, we considered that it may not return to a higher dielectric constant because the unbridged materials were removed [17]. It is assumed that Si–O groups made by UV treatment may change to Si–CH_3_ groups included in unbridged material because of stability. However, in the case of washed PDMS, unbridged materials were already removed. Therefore, it can be said that Si–O groups cannot change to Si–CH_3_ groups and washed PDMS may not return to a higher dielectric constant. In practical use, the sensor would be mounted onto the endoscope as a disposable part, which would require a relatively short lifetime of the sensor. 

In this experiment, we found that the fluctuation of the dielectric constant depended on humidity. The fluctuation of the washed samples appeared to be smaller than that of the non-washed samples. We considered that this originated from the surface property of the samples. It is known that a polymer with many hydrophilic functional groups is easily affected by humidity [23,24,25]. After the UV treatment, the PDMS surface became hydrophilic. Washing with the buffer turned the surface back to hydrophobic, culminating in robustness against humidity and thus small fluctuations.

### 3.2. Sensor Characteristics

**Figure 10:**
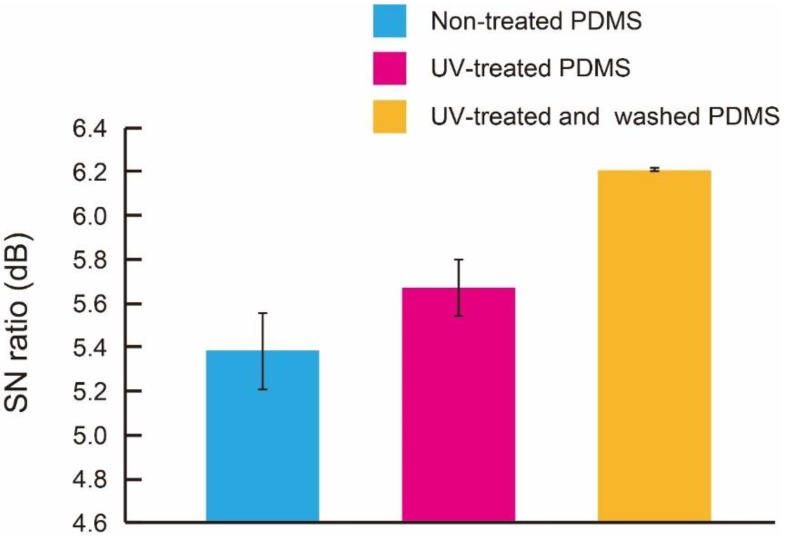
SN ratio obtained from nine devices, the nontreated devices, UV-treated devices, and the UV-exposed and washed devices (n = 3).

The capacitive force sensor was manufactured using PDMS membranes treated with UV for 300 s and washed with a buffer for 10 s. The manufacturing process took 6 h, therefore the low dielectric constant was considered to be maintained. Figure 10 shows the SN ratio of the sensor using PDMS membranes that underwent no treatment, UV-treatment, and a combination of UV treatment and washing. The UV treatment increased the SN ratio from 5.4 to 5.6 in dB, the washing treatments increased the SN ratio from 5.6 to 6.2 in dB, i.e., approximately 1.2 times. The wiring part had parasitic capacitance because parasitic capacitance is caused by close wiring. Therefore, we replaced layers 6 and 7 with low-dielectric PDMS layers in order to reduce parasitic capacitance. It can be said that we can successfully reduce parasitic capacitance by stacking multiple thin low-dielectric PDMS layers. This result indicates that treatment of PDMS with UV and washing reduces the dielectric constant of the PDMS membrane structures and thus improves the performance of the capacitive sensor. 

## 4. Conclusions

We experimentally confirmed that the furthest reduction of dielectric constant of PDMS achieved by the combination of treatment and washing with the ethanol-toluene buffer solution for the first time. We applied the proposed combination to the 3-D flexible sensor that we reported in our prior work and successfully enhanced the SN ratio from 5.4 to 6.2 dB. The proposed technique is simple but effective and can be readily applicable to PDMS-based flexible sensors.
